# Pheochromocytoma and paraganglioma: germline genetics and hereditary syndromes

**DOI:** 10.1530/EO-22-0044

**Published:** 2022-06-28

**Authors:** Christie G Turin, Molly M Crenshaw, Lauren Fishbein

**Affiliations:** 1 Department of Medicine, Division of Endocrinology, Metabolism and Diabetes, University of Colorado, Aurora, Colorado, USA; 2 Department of Pediatrics, Combined Pediatrics-Medical Genetics Residency Program, University of Colorado, Aurora, Colorado, USA; 3 Division of Biomedical Informatics and Personalized Medicine, University of Colorado School of Medicine, Aurora, Colorado, USA

**Keywords:** pheochromocytoma, paraganglioma, hereditary genetics, hereditary syndromes

## Abstract

Pheochromocytomas (PCCs) and paragangliomas (PGLs) are neuroendocrine tumors arising from the adrenal medulla and extra-adrenal ganglia, respectively. Approximately 15–25% of PCC/PGL can become metastatic. Up to 30–40% of patients with PCC/PGL have a germline pathogenic variant in a known susceptibility gene for PCC/PGL; therefore, all patients with PCC/PGL should undergo clinical genetic testing. Most of the susceptibility genes are associated with variable penetrance for PCC/PGL and are associated with different syndromes, which include susceptibility for other tumors and conditions. The objective of this review is to provide an overview of the germline susceptibility genes for PCC/PGL, the associated clinical syndromes, and recommended surveillance.

## Introduction

Pheochromocytomas (PCCs) and paragangliomas (PGLs) are rare neuroendocrine tumors that are derived from the adrenal medulla and extra-adrenal sympathetic or parasympathetic ganglia, respectively. PCC/PGL are estimated to occur in 2–8 per million individuals (Lloyd 2017). PCC/PGL can cause high morbidity from catecholamine secretion and mass effect and can lead to high mortality if unrecognized or when metastatic. About 15–25% of PCC/PGL metastasize (Ayala-Ramirez *et al.* 2011), and once this happens, there is a 43–69% 5-year survival rate (Ayala-Ramirez *et al.* 2011, Fishbein *et al.* 2017, Hamidi *et al.* 2017). Metastatic disease can be found at the time of initial diagnosis or even over 20 years later. Most head and neck PGLs are from parasympathetic ganglia and do not secrete catecholamines; in contrast, PCCs and most abdominal/pelvic PGLs produce and secrete catecholamines.

PCC/PGLs are a rare secondary cause of hypertension, accounting for ~0.3% of cases (Anderson *et al.* 1994). It is estimated that PCC/PGLs are the etiology for less than 2% of primary pediatric hypertension cases (Wyszynska *et al.* 1992). Untreated disease can lead to significant cardiovascular morbidity and mortality; therefore, despite being rare, PCC/PGL should be considered in adults and children with uncontrolled hypertension and/or those undergoing work-up for secondary causes of hypertension, even in the absence of the classic triad (sweating, palpitations and headaches) (Cohen *et al.* 2006).

PCC/PGL should be suspected in patients with hyperadrenergic symptoms, early-onset or resistant hypertension, new hypertension and new or worsening diabetes, known familial syndromes or tumors related to familial syndromes, and incidental adrenal adenomas that are lipid poor on CT (non-contrast Hounsfield units greater than 10) or MRI (no loss of signal intensity on in and out of phase imaging). When suspected, biochemical evaluation should include measurement of plasma-free metanephrines or 24-h urinary fractionated metanephrines and catecholamines. Both plasma and 24-h urine tests have similar sensitivity and specificity (Lenders *et al.* 2014). Measurement of age-adjusted plasma metanephrines in a supine position could reduce the false-positive rate (Kline *et al.* 2021), although this may not be always feasible as a first screening step. Abnormal results are at least two to four times above the upper limit of normal. Results above the upper limit of the normal range but less than two times elevated are considered indeterminate. In these indeterminate cases, the first step is to look for potentially interfering factors (Lenders *et al.* 2014). In our experience, potential causes include medications (e.g. tricyclic antidepressants, selective serotonin reuptake inhibitors, serotonin and norepinephrine reuptake inhibitors, alpha-adrenergic blockers, etc.), drugs (e.g. cocaine, marijuana, stimulants, caffeine, etc.), or position (i.e. sitting up rather than lying down for the plasma tests). Once tumoral catecholamine production is confirmed, cross-sectional imaging to localize the tumor is the next step, typically starting with the abdomen/pelvis as 80–85% of PCC/PGL will be in this region (Lenders *et al.* 2014). When planning for surgical resection, patients should undergo alpha blockade prior to resection. The use of alpha blockade and modern anesthesia have reduced surgical morbidity and mortality (Bruynzeel *et al.* 2010). If metastatic disease is suspected, functional imaging with DOTATATE PET/CT or MIBG scans can be useful (Fishbein *et al.* 2021). Any patient who has a history of PCC/PGL or a known germline susceptibility gene pathogenic variant (PV) for PCC/PGL should be screened prior with plasma free metanephrines to pregnancy or immediately upon becoming pregnant to prevent poor maternal and fetal outcomes from unrecognized PCC/PGL (Bancos *et al.* 2021).

About 30–40% of the PCC/PGL cases are associated with a germline PV in a known susceptibility gene (Fishbein *et al.* 2013), so it is important that patients with PCC/PGL be offered clinical genetic testing. Providers must be aware of the high rate of hereditary PCC/PGL and make the referral for all patients to have genetic counseling and possible testing. If the patient carries a PCC/PGL susceptibility gene, providers must be knowledgeable about the recommendations for gene-specific screening and surveillance for the associated syndromes (Table 1). Here, we provide an updated overview of the germline susceptibility genes and the associated clinical syndromes.

**Table 1:** PCC/PGL syndromes and screening recommendations.

Syndrome	Associated syndromic features	Recommended first screening	Recommended follow-up screening
Von Hippel Lindau	CNS/retinal hemangioblastoma, renal cell carcinoma, pheochromocytoma, pancreatic neuroendocrine tumor, endolymphatic sac tumors, renal and pancreatic cysts	5 years old: plasma-free metanephrines or 24-h urine-fractionated metanephrines, audiology, ophthalmology, blood pressure, neurologic exam.	Yearly repeat initial screening. At 11 years, begin MRI brain and spinal cord every 2 years. At age 15, begin MRI abdomen every 2 years.
Multiple endocrine neoplasia type 2	MEN2A: Medullary thyroid cancer, pheochromocytoma, primary hyperparathyroidism MEN2B: Medullary thyroid cancer, pheochromocytoma, mucosal neuromas	MEN 2A: Calcitonin at 3–5 years old or prophylactic thyroidectomy. Calcium and PTH at 8 years old or 20 years old depending on variant. MEN 2B: Calcitonin at 6 months old or prophylactic thyroidectomy. Plasma-free metanephrines or 24-h urine-fractionated metanephrines at 11 years old (for PVs in codon 634, 883, or 918) or 16 years old (for other PVs).	Metanephrines, calcitonin, calcium and PTH yearly. Imaging indicated if labs abnormal.
Neurofibromatosis type 1	Neurofibromas (cutaneous or subcutaneous), plexiform neurofibromas, optic glioma, malignant peripheral nerve sheath tumor, café-au-lait spots, freckling, long bone abnormalities. Associated: pheochromocytoma, juvenile chronic myelogenous/myelomonocytic leukemia	At diagnosis: dermatology, ophthalmology, developmental assessment, blood pressure. If elevated blood pressure or symptoms of catecholamine excess, measure plasma-free metanephrines or 24-h urine-fractionated metanephrines. Mammography at age 30.	Yearly physical exam, ophthalmology evaluation of MRI brain if symptomatic. Mammography annually after 30 years.
*SDHx-*associated hereditary paraganglioma-heochromocytoma syndrome	*SDHA, B, C, D*: Paraganglioma, pheochromocytoma at any location, renal cell carcinoma, GI stromal tumors *SDHAF2*: Head/neck paraganglioma	SDHB: 6–10 years old plasma-free metanephrines or 24-h urine-fractionated metanephrines and full- body MRI from skull base to pelvis. SDHA, C, D: 10–5 years old plasma-free metanephrines or 24-h urine-fractionated metanephrines and full-body MRI from the skull base to pelvis. *SDHAF2*: less well-defined. Typically use above recommendations.	<18 years old: Biochemical evaluation every 2 years and full-body MRI from skull base to pelvis every 2–3 years. >18 years old: annual biochemical evaluation and full-body imaging from skull base to pelvis every 2–3 years.
*TMEM127-*associated PCC/PGL	Paraganglioma, pheochromocytoma, renal cell carcinoma	At diagnosis: Plasma-free metanephrines or 24-h urine-fractionated metanephrines and full-body MRI from skull base to pelvis.	Annual biochemical evaluation and full-body MRI from skull base to pelvis every 2 years.
*MAX-*associated PCC/PGL	Pheochromocytoma, paraganglioma	At diagnosis: Plasma-free metanephrines or 24-h urine-fractionated metanephrines and full-body MRI from skull base to pelvis.	Annual biochemical evaluation and full-body MRI from skull base to pelvis every 2 years.

## Germline genetics and clinical syndromes

Over the past two decades, there have been remarkable advances in identifying hereditary causes of PCC/PGL that have led to a better understanding and improvement in the management and surveillance of patients. Up to 30–40% of affected patients carry a germline PV in one of over 12 well-defined susceptibility genes (Fishbein *et al.* 2013). Interestingly, even patients with apparently sporadic PCC/PGL (meaning a single PCC or PGL and no family history) have a frequency of germline PVs in a known susceptibility gene of approximately 11–13% (Fishbein *et al.* 2013, Brito *et al.* 2015). Therefore, expert guidelines recommend performing clinical genetic testing in all patients with PCC/PGL, regardless of age, family history, or clinical features (Lenders *et al.* 2014, Fishbein *et al.* 2021). If a PV in a susceptibility gene is identified, the patient should be screened for additional primary PCC/PGL, recurrence, and the other associated conditions. In addition, blood relatives should be offered cascade clinical genetic testing as they may be asymptomatic PV carriers. There are some genotype/phenotype correlations for PCC/PGL risk with each gene (Table 2) which can help guide screening and surveillance.

**Table 2:** Clinical characteristics associated with PCC/PGL susceptibility genes.

PCC/PGL susceptibility gene	Transmission	Penetrance for PCC/PGL	Adrenal PCC	HNPGL	Extra-adrenal PGL	Risk of multifocal primary PCC/PGL	Risk of metastatic disease
*VHL*	Autosomal dominant	19% 60% for type 2	+++	+	+	+++	<5%
*RET*	Autosomal dominant	50%	+++	+	+	+++	<5%
*NF1*	Autosomal dominant	7.7–14%	+++	+	+	++	~12%
*SDHB*	Autosomal dominant	22–26% by age 60	++	++	+++	++	25–50%
*SDHD*	Autosomal dominant – paternal inheritance	43% by age 60	++	+++	++	+++	3–8%
*SDHC*	Autosomal dominant	25% by age 60^a^	+	+++	+	++	<5%
*SDHA*	Autosomal dominant	10% by age 70	++	++	++	+	12%^a^
*SDHAF2*	Autosomal dominant – paternal inheritance	Ukn^b^	Ukn^b^	+++	Ukn^b^	+++	<5%
*TMEM127*	Autosomal dominant	Ukn^b^	+++	+	+	++	<5%
*MAX*	Autosomal dominant	Ukn^b^	+++	Ukn^b^	+	+++	<10%^a^

+++, very likely; ++, likely; +, less likely but can occur. ^a^Small sample size may skew estimates. ^b^Ukn, unknown.

**Figure 1:**
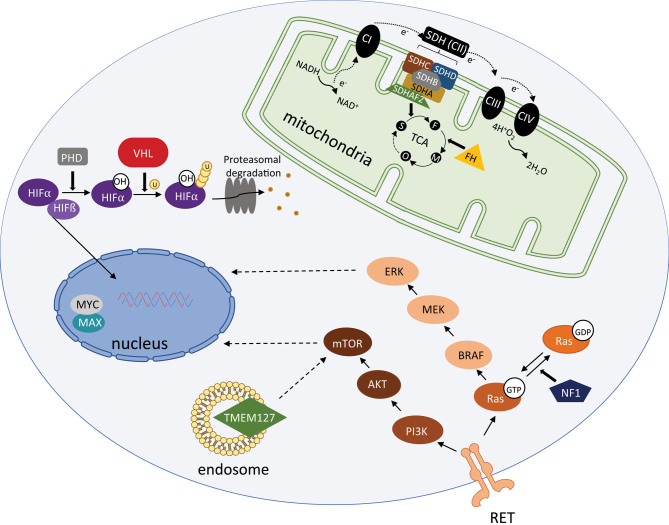
Germline PCC/PGL susceptibility gene cellular pathways.

Germline susceptibility genes for PCC/PGL are involved in a broad range of pathways within the cell and in general can be categorized into two expression clusters: pseudohypoxia (cluster 1) and kinase signaling (cluster 2) (Dahia 2014). Cluster 1, or the pseudohypoxia cluster, includes genes that are involved in metabolic or oxygen-sensing pathways. The genes included in this cluster are *SDHx* *, VHL, FH,* and* EPAS1*. Cluster 2, or the kinase signaling cluster, includes genes involved in kinase signaling such as *RET*, *NF1, MAX,* and *TMEM127*. Figure 1 further illustrates the cellular pathways involved, which are further discussed in the sections below. Somatic mutations in other genes create additional expression clusters not discussed here, as this is outside the scope of the current review on hereditary predisposition.

## Von Hippel-Lindau syndrome

Von Hippel-Lindau syndrome (VHL) is caused by inactivating PVs in the von Hippel-Lindau (*VHL*) gene, located on chromosome 3p25.3 (OMIM: 608537). The *VHL* gene consists of three exons and encodes two isoforms of the tumor suppressor protein (pVHL_30_ and pVHL_19_). The normal function of pVHL is to bind to Hypoxia-inducible factor (HIF) alpha under normoxic conditions, promoting its ubiquitination and subsequent proteasomal degradation. This prevents HIF from translocating to the nucleus and activating the transcription of hypoxia-inducible genes. Under hypoxic conditions, or if there are PVs in the *VHL* gene, pVHL does not bind to HIF. The disruption of this interaction allows HIF to avoid degradation and instead move to the nucleus, leading to dysregulated activation of the hypoxic response, thereby promoting tumorigenesis.

VHL is an autosomal dominant hereditary cancer syndrome characterized by the development of a variety of tumors such as hemangioblastomas of the CNS including the retina, renal cell carcinoma (RCC), pancreatic neuroendocrine tumors, PCC, endolymphatic sac tumors, and visceral cysts (including renal and pancreatic). It affects approximately 1 in 36,000 individuals and has a 95% penetrance by age 60 (Maher *et al.* 1991). Patients with VHL syndrome are divided into two types based on the risk of developing PCC. Patients with type 1 VHL syndrome (typically those with protein-truncating variants) have a lower risk of developing PCC and lower risk of developing RCC. Patients with type 2 disease (typically those with missense variants) have a higher risk of developing PCC. Type 2 disease is further subdivided into three groups based on the risk of developing RCC (high, low, and no risk for RCC) (Knauth *et al.* 2006).

A recent systemic review and meta-analysis that included 4263 patients with VHL across 45 studies showed a frequency of PCC/PGL of 19.4% overall, but that frequency increased to 60% in patients with type 2 VHL (Castro-Teles *et al.* 2021). Furthermore, this study suggested that of patients with VHL who develop PCC, almost 60% have bilateral PCCs. The mean age of diagnosis of PCC is 29 years old. This meta-analysis did not assess for risk of metastatic disease; however, prior studies suggest the risk of metastatic PCC is about 5% (Maher *et al.* 2011). PGLs are rare but can occur (Boedeker *et al.* 2009, Gaal *et al.* 2009). Most VHL-associated PCC/PGL are normetanephrine predominant, due to a lack of expression of phenylethanolamine-N-methyltransferase, the enzyme that converts norepinephrine to epinephrine (Eisenhofer *et al.* 2017). Given the high prevalence and early-onset of PCC/PGL for those with VHL, guidelines recommend screening for PCC/PGL starting at age 5 with annual lifelong biochemical testing (VHL Alliance 2015, Rednam *et al.* 2017). Screening for the other VHL manifestations and tumors is outlined in Table 1.

## Multiple endocrine neoplasia type 2

Multiple endocrine neoplasia type 2 (MEN2) is caused by activating PVs in the *RET (*rearranged during transfection) gene. The *RET* proto-oncogene is located on chromosome 10q11.21 (OMIM: 164761). Its product is a transmembrane receptor tyrosine kinase that activates downstream PI3K-AKT and MAPK-ERK kinase signaling pathways, leading to cellular proliferation and decreased apoptosis.

MEN2 is an autosomal dominant hereditary tumor syndrome that affects 1 in 30,000 individuals and is classified into two categories: MEN2A (95% of cases) and MEN2B (Wells *et al.* 2015). Patients with MEN2A show a high penetrance for medullary thyroid carcinoma (MTC) or C-cell hyperplasia (nearly 100%), PCC (50%), and multiglandular parathyroid hyperplasia or adenoma (20–30%), leading to primary hyperparathyroidism (Wells *et al.* 2015). Patients with MEN2B, primarily caused by a missense variant in codon 918, present with MTC (100%) and PCC (~50%), a marfanoid habitus, mucosal neuromas (Wells *et al.* 2015). Those with MEN2B typically do not develop primary hyperparathyroidism. Overall, the mean age at diagnosis of PCC is about 30–40 years old (Castinetti *et al.* 2017). Of the patients who develop PCC, about 50% will have bilateral disease. The risk of metastatic disease is low at less than 5%. Most MEN2-associated PCC produce metanephrine predominantly (Eisenhofer *et al.* 2011).

The risk of developing PCC varies depending on the *RET* codon affected, so the recommended age to start screening also depends on the specific codon affected. It is recommended to start screening for PCC at age 11 in patients with high risk (codon 634, 883) and highest risk (codon 918) variants due to higher penetrance of PCC, and at age 16 for those with other *RET* variants (Wells *et al.* 2015). Screening for PCC involves measuring plasma-free or 24-h urine-fractionated metanephrines. Imaging is only recommended if biochemical testing is elevated. Screening for the other manifestations for MEN2 are described in Table 1.

## Neurofibromatosis type 1

Neurofibromatosis type 1 (NF1) is caused by inactivating PVs in the *NF1* gene. The *NF1* gene is located on chromosome 17q11.2 (OMIM: 613113). It is one of the largest genes in the human genome, with 60 exons spanning over 350 kb of genomic DNA. The *NF1* gene encodes the protein neurofibromin, with two major isoforms, neurofibromin type I and II. Neurofibromin is a tumor suppressor that acts as a RAS GTPase to downregulate the MAPK signaling cascade. Loss of function variants in the *NF1* gene leads to uncontrolled cellular proliferation and tumorigenesis.

NF1 is an autosomal dominant tumor-predisposition syndrome. NF1 affects ~1 in 2000–3500 individuals (Legius *et al.* 2021). This syndrome is characterized by the presence of two or more of the following recently revised NIH consensus criteria: six or more café-au-lait spots over 5 mm in greatest diameter in pre-pubertal patients or over 15 mm in greatest diameter in post-pubertal patients; two or more neurofibromas of any type or one plexiform neurofibroma; freckling in the axillary or inguinal regions; optic glioma; two or more Lisch nodules (benign iris hamartomas); a distinctive bone lesion such as sphenoid dysplasia, anterolateral bowing of the tibia, or pseudoarthrosis of a long bone; or heterozygous pathogenic *NF1* variant; or a parent who meets the diagnostic criteria listed above (Legius *et al.* 2021).

The prevalence of PCC in those with NF1 ranges from 7.7 to 14% depending on the study (Zinnamosca *et al.* 2011, Képénékian *et al.* 2016). The risk of metastatic PCC is about 12% (Bausch *et al.* 2006). Mean age at diagnosis of PCC is 41 years. NF1-associated PCC tend to have metanephrine predominance (Eisenhofer *et al.* 2011). Guidelines recommend screening patients with NF1 and hypertension, those older than 30 years old, pregnant patients, and/or symptomatic patients (Stewart *et al.* 2018). However, given the suspected higher prevalence and cardiovascular risks if detection is delayed, some authors suggest early and frequent screening with biochemical testing every 3 years for all patients with NF1 (Gruber *et al.* 2017, Al-Sharefi *et al.* 2019).

## Hereditary paraganglioma-pheochromocytoma syndromes

Hereditary paraganglioma-pheochromocytoma syndromes are caused by inactivating PVs in the succinate dehydrogenase subunit (*SDH*) genes (*SDHx*)leading to defects in the assembly of the SDH protein complex of the Kreb’s cycle and the mitochondrial respiratory chain (complex II). This disruption of the SDH complex leads to increased succinate levels, which in turn inactivates prolyl hydroxylases causing stabilization of HIFs. *SDHA, SDHB, SDHC,* and *SDHD* genes encode the four subunits of SDH. There are several co-factors for the SDH complex, of which one, SDHAF2, has been shown to be associated with PCC/PGL as well. SDH is a respiratory chain enzyme that catalyzes the oxidation of succinate to fumarate and links the Krebs cycle with the electron transport chain in the mitochondria. The subunits SDHA and SDHB are in the mitochondrial matrix, anchored to the inner membrane by subunits SDHC and SDHD. All of the *SDHx*-associated Hereditary Paraganglioma-Pheochromocytoma Syndromes are autosomal dominant in inheritance.

Approximately 20% of patients with PCC/PGL have a PV in one of the *SDHx* genes, with *SDHB* and *SDHD* PVs being the most commonly associated. Most *SDHx* *-*related PCC/PGL have normetanephrine and/or dopamine predominant production (Eisenhofer *et al.* 2011), and some are non-secretory, especially those in the head and neck. Patients with germline PVs in *SDHx* genes are at higher risk than the general population for RCC (8% or less risk) and gastrointestinal stromal tumors (very low risk but elevated over the general population) (Rednam *et al.* 2017).

A recent international consensus statement on screening and monitoring of asymptomatic *SDHx* PV carriers (Amar *et al.* 2021) slightly altered the previous guidelines on screening for those with *SDHx* PVs. The prior guidelines recommended that screening should begin between ages 6 and 8 for all *SDHx* carriers and should include annual biochemical testing and full-body MRI from the skull base to the pelvis every 2 years (Rednam *et al.* 2017). The 2021 international consensus guidelines suggest a variation on this. Given the higher risk for metastatic disease in patients with *SDHB* PVs and reported cases of early-onset of disease during childhood, it is proposed to start screening at age 6–10 for asymptomatic *SDHB* PV carriers and at age 10–15 for carriers of PVs in *SDHA, SDHC,* and* SDHD* genes (Amar *et al.* 2021). If initial screening is negative, biochemical testing should be performed at least every 2 years during childhood (i.e. age <18) and every year during adulthood, and full-body MRI from the skull base to pelvis should be performed every 2–3 years from the time the variant is known. Some suggest that a ^68^Ga-DOTATE PET/CT scan can be used instead of MRI if available, but it is important to note the increased cost, at least in the United States, and the increased radiation exposure. Of note, they do not recommend a different interval for screening based on the variable penetrance of PCC/PGL depending on the subunit affected. This is an area for future research and clinical guidelines.

Below we will discuss some *SDHx* gene-specific disease-related details.

## *SDHB-*associated hereditary paraganglioma-pheochromocytoma syndrome

Among the hereditary paraganglioma-pheochromocytoma syndromes, *SDHB-*associated PCC/PGL are the most common. The *SDHB* gene is located on chromosome 1p36.13 (OMIM: 185470), and it encodes an iron-sulfur subunit of the SDH complex. PVs in *SDHB* confer susceptibility for PCC/PGL in an autosomal dominant fashion.

Initial penetrance rates for *SDHB* PVs were believed to be almost 100% in a lifetime (Ricketts *et al.* 2010), but these data came mostly from probands. More recent studies looking at non-proband relatives suggest penetrance is much lower, around 22–26% by age 60 (Jochmanova *et al.* 2017, Andrews *et al.* 2018). If both probands and non-probands are included, the penetrance of PCC/PGL by age 60 ranges from 24 to 58% (Jochmanova *et al.* 2017, Andrews *et al.* 2018). Interestingly, there may be higher age-related penetrance in male patients than in female patients (Jochmanova *et al.* 2017, Andrews *et al.* 2018).

Most *SDHB*-associated PCC/PGL are extra-adrenal in the abdomen and pelvis, but they can develop in the adrenal gland and in the head and neck as well. *SDHB*-associated PCC/PGL have the highest rates of developing metastatic disease, with estimates of about 25–50% depending on the study (Jochmanova *et al.* 2017, Niemeijer *et al.* 2017). There are some caveats with interpreting risk of metastatic disease for carriers of *SDHB* PVs because the definition of metastatic PCC/PGL has not been consistent in the literature. The current WHO definition of metastatic disease is tumor in sites without chromaffin tissues or ganglia such as lymph nodes, bone, and brain (Lloyd 2017). Nevertheless, clinically, the most common sites of metastatic disease include bone, liver, lung, and lymph nodes. Technically, the liver and lung have ganglia, and therefore, lesions seen in these tissues can be either multifocal primary paraganglioma or metastatic disease; however, clinically, these are treated the same (Fishbein *et al.* 2021). Carriers of *SDHB* PVs are at risk for RCC (4–5% risk by age 60) and have an increased risk to develop gastrointestinal stromal tumors compared with the general population (Andrews *et al.* 2018).

## *SDHD*-associated hereditary paraganglioma-pheochromocytoma syndrome

*SDHD*-associated PCC/PGL are the second most commonly seen. The *SDHD* gene is located on chromosome 11q23.1 (OMIM: 602690) and encodes the subunit with a ubiquinone binding site to transfer electrons from the iron-sulfur clusters in the SDHB subunit of the enzyme complex. The SDHD subunit helps to anchor the protein complex to the inner mitochondrial membrane. PVs in *SDHD* are inherited in an autosomal dominant pattern with paternal inheritance. Initially, it was believed that *SDHD* was maternally imprinted, but that has been difficult to prove, and some tissues have biallelic expression of *SDHD* (Yamashita *et al.* 2009). In addition, there are rare case reports of maternal transmission of *SDHD* (Pigny *et al.* 2008, Yeap *et al.* 2011). The paternal inheritance, and also the rare exceptions, make counseling on risk and analysis of family history more difficult.

The penetrance for PCC/PGL in *SDHD* carriers is around 43.2% by age 60 (Andrews *et al.* 2018). Patients with *SDHD*-associated PGL often have multifocal primary PCC/PGL. More than half of patients (58%) will have head and neck PGLs by the median age of 40 and 17% will have other PCC/PGL by the median age of 23 (Andrews *et al.* 2018). The risk of metastatic disease is between 3 and 8% (Van Hulsteijn *et al.* 2012, Andrews *et al.* 2018). Individuals with *SDHD* PVs have an increased risk for RCC (estimated at 1%) (Andrews *et al.* 2018) and gastrointestinal stromal tumors.

## *SDHC*-associated hereditary paraganglioma-pheochromocytoma syndrome

*SDHC*-associated PCC/PGL are not as common as *SDHB* or *SDHD*-associated tumors. The *SDHC* gene is located on chromosome 1q23.3 (OMIM: 602413). Its product is one of the membrane proteins that anchor other subunits of the complex to form the catalytic core. PVs in *SDHC* are inherited in an autosomal dominant fashion.

In a review of the literature describing a total of 62 patients with *SDHC-*related PCC/PGL, 81% of them had head and neck tumors (all were non-functional), 10% had tumors located in the mediastinum, and 1.3% in the adrenal glands (Else *et al.* 2014). Among these patients, 23% had multiple primary PCC/PGL. Only 2.5% of patients had metastatic disease.

Only a few studies have looked at penetrance in *SDHC* carriers for PCC/PGL. One study of 34 *SDHC* carriers found that 56% (19 of 34) were affected (Andrews *et al.* 2018). The penetrance for PCC/PGL in the non-probands (*N* = 26) was 25%, but clearly, the small sample size is a limitation to the study. Another recent retrospective case series that included 91 patients (46 probands) with confirmed *SDHC* PVs revealed that PCC/PGL incidence in non-probands by age 60 was 6.7% (Williams *et al.* 2022). Of importance, no large study evaluating asymptomatic relatives who carry this germline PV has been done. In general, it is estimated that the penetrance of *SDHC* PVs for PCC/PGL is low given the lack of tumors in first-degree relatives overall, but exact penetrance is difficult to determine given the small number of patients studied. *SDHC* PVs are associated with GIST (6 of 91 or 6.6%) and rarely with RCC (Williams *et al.* 2022).

## *SDHA-*associated hereditary paraganglioma-pheochromocytoma syndrome

*SDHA*-associated PCC/PGL are rare and data are limited by small cohorts. The *SDHA* gene is located on chromosome 5p15.33 (OMIM: 600857) and encodes the subunit of SDH which contains a covalently attached flavin adenine dinucleotide co-factor and acts as a catalytic subunit. Biallelic PVs in the *SDHA* gene are reported as the cause of Leigh syndrome, which is an early-onset neurodegenerative disorder (Horváth *et al.* 2006). In 2010, the first report of a patient with PCC/PGL and an autosomal dominantly inherited heterozygous germline PV in *SDHA* was published (Burnichon *et al.* 2010).

A retrospective study revealed that 7.6% of patients (30 of 393) with PCC/PGL, who had no other hereditary cause of the tumor, had a germline PV in *SDHA* (Van der Tuin *et al.* 2018). Median age at diagnosis was 43 years old and penetrance of *SDHA*-related disease in non-probands was only 10% at age 70 years (Van der Tuin *et al.* 2018). Although the penetrance is low, the rate of metastatic disease for those with *SDHA*-associated PCC/PGL is high at 12% (Bausch *et al.* 2017). *SDHA* PVs are associated with GIST and RCC as well, both at low penetrance.

Although the consensus guidelines still recommend biochemical evaluation annually and full-body imaging every 2–3 years (Amar *et al.* 2021), these recommendations are being questioned by experts given the very low penetrance of *SDHA* PVs. As large gene panels are being used more frequently for susceptibility testing in individuals with other cancers, it is becoming more common to find incidental PVs and likely PVs in *SDHA* with unclear significance. More research is needed to understand the true penetrance of disease and what optimal screening is necessary.

## *SDHAF2-*associated hereditary paraganglioma-pheochromocytoma syndrome

The *SDHAF2* (also called *SDH5)* gene is located on chromosome 11q12.2 (OMIM: 613019). SDHAF2 is one of the co-factors of the SDH complex and the only co-factor currently associated with increased risk of PCC/PGL. The protein is needed for flavination of the SDHA subunit which is required for the protein complex activity. PVs in *SDHAF2* have an autosomal dominant inheritance with a paternal transmission, similar to *SDHD*.

Variants in *SDHAF2* are rare in the PCC/PGL population as a whole. A multicenter study evaluated 315 patients with PCC/PGL, who had no known other susceptibility gene PV, for germline variants in *SDHAF2* and did not identify any variants in this gene (Bayley *et al.* 2010). Another study that included patients from a population-based registry analyzed samples of 972 registrants with PCC/PGL who did not have PVs in the common susceptibility genes, *RET, VHL, SDHB, SDHC, SDHD,* and*NF1,* to determine the presence of genetic variants in the more rare susceptibility genes (including *SDHA, MAX, TMEM127,* and *SDHAF2*). Of the 972 subjects, 58 patients had a PV in one of the rare susceptibility genes and only one was in *SDHAF2* (Bausch *et al.* 2017). Much of the clinical information comes from one large family of 57 individuals where 24 had an *SDHAF2* PV (Kunst *et al.* 2011). The age of onset for PCC/PGL was 33 years, and most patients had tumors in the head and neck area (88%), and 91% were multifocal. There was no evidence of metastatic disease in this cohort.

## Other syndromes associated with hereditary PCC/PGL

### *TMEM127*-associated PCC/PGL

The *TMEM127* gene is located on chromosome 2q11.2 (OMIM: 613403), and it encodes a protein called transmembrane protein 127 that works as a negative regulator in the mTOR signaling pathway. *TMEM127* PVs inherited in an autosomal dominant manner increase risk for PCC/PGL. However, penetrance appears to be low as germline PVs in *TMEM127* account for a small percentage of all PCC/PGL. The initial study found that 1% of apparently sporadic PCC/PGL were from patients with germline PVs in *TMEM127* (Qin *et al.* 2010). Bausch *et al.* evaluated the clinical characteristics and penetrance of patients with PCC/PGL who did not have classic known susceptibility gene PVs. Of the 972 cases, 21 (2.1%) had a germline PV in *TMEM127* (Bausch *et al.* 2017).

A recent study included 110 index patients with *TMEM127* germline PVs and evaluated the tumor characteristics of this cohort (Armaiz-Pena *et al.* 2021). The majority of patients had PCC (85.5%), and approximately 9% of patients had PGL. Multifocal primary tumors were described in 33% of patients and metastatic disease occurred in 2.8%. RCC was found in 5.4% of the cohort (6 of 110). The average age of presentation was 45 years old (Armaiz-Pena *et al.* 2021). Guidelines suggest similar screening to those with *SDHx* PVs with annual biochemical testing and MRI from the skull base to pelvis every 2 years (Rednam *et al.* 2017).

### *MAX*-associated PCC/PGL

The *MAX* gene is located on chromosome 14q23.3 (OMIM: 154950) and it encodes MYC-associated protein X (MAX). This protein plays a fundamental role in regulating MYC activity which affects cell differentiation and growth. PVs in *MAX* are inherited in an autosomal dominant pattern and increase the risk of PCC/PGL. Germline PVs in *MAX* have been identified in about 1% of PCC/PGL without other known susceptibility gene variants (Burnichon *et al.* 2012, Bausch *et al.* 2017). The majority of reported cases are adrenal PCC with over half having bilateral disease. The rate of metastatic disease is difficult to define given the small cohorts but may be increased as one study had 2 of 23 cases (8.7%) having metastases (Burnichon *et al.* 2012). Guidelines suggest similar screening to those with *SDHx* PVs with annual biochemical testing and full-body MRI imaging from the skull base to the pelvis every 2 years (Rednam *et al.* 2017).

## Additional associations

### Hereditary leiomyomatosis and renal cell carcinoma syndrome

Hereditary leiomyomatosis and renal cell carcinoma syndrome (HLRCC) is an autosomal dominant syndrome caused by inactivating PVs in the fumarate hydratase (*FH*)gene. The *FH* gene is located on chromosome 1q43 (OMIM: 136850) and encodes the fumarate hydratase enzyme that converts fumarate to malate in the Krebs cycle. Inactivating *FH* variants lead to increased levels of fumarate, which inactivates prolyl hydroxylases causing stabilization of the HIF 1 pathway. Heterozygous PVs in *FH* lead to autosomal dominant HLRCC. Biallelic PVs in *FH* lead to autosomal recessive fumarate hydratase deficiency, an early-onset metabolic disorder.

HLRCC is most commonly associated with cutaneous leiomyomas (73%) and uterine leiomyomas (77%) and RCC (around 19%) (Muller *et al.* 2017). One of the largest studies, describing 182 patients with germline PVs in *FH*, found the frequency of PCC/PGL was around 1% (2 of 182 patients in the cohort) and interestingly PCC/PGL have not been identified in patients with other features of HLRCC (Muller *et al.* 2017). One caveat of this study was that not all patients were screened for PCC/PGL with biochemical testing or whole-body MRI. Most, if not all, did have at least abdominal MRIs to screen for RCC. Given the rarity of *FH*-associated PCC/PGL, the mean age is difficult to assess, as is the risk of metastatic disease, although it is believed to potentially be high (Castro-Vega *et al.* 2014). Screening recommendations for PCC/PGL in those with HLRCC are not well established. Most recommend at least baseline full-body MRI from the skull base to pelvis and plasma metanephrines at the time of variant identification. The interval for repeating the testing is not well defined.

### Polycythemia and paraganglioma syndrome

Germline *EPAS1* PVs lead to hereditary erythrocytosis syndrome. The *EPAS1* gene is located on chromosome 2p21 (OMIM: 603349). It encodes the endothelial PAS domain-containing protein 1, also known as HIF 2α. HIF proteins are important factors regulating hypoxia-inducible gene pathways in the cell involved in energy metabolism, iron metabolism, angiogenesis, and erythropoiesis. Interestingly, gain-of-function somatic mosaic *EPAS1* PVs associated with PCC/PGL were first described in 2012 (Zhuang *et al.* 2012), and subsequently, there was another report of multifocal primary PCC/PGLs in a patient having the same somatic *EPAS1* PV in the tumors which was not seen in the germline DNA (Comino-Méndez *et al.* 2013). Patients with somatic mosaic *EPAS1* variants tend to develop PCC/PGL and polycythemia and may have an increased risk for somatostatinoma (Zhuang *et al.* 2012).

### Newly identified potential susceptibility genes

Despite the high number of known PCC/PGL susceptibility genes, there are still some families with multiple affected individuals with PCC/PGL, or with individuals with multifocal primary PCC/PGL, without an identified heritable cause; therefore, further investigations for additional susceptibility genes are ongoing. Genomic evaluation of some of these familial cases or multifocal primary tumors have identified several putative additional susceptibility genes with varying levels of evidence. Another Krebs cycle enzyme has been implicated, the *MDH2* gene which encodes the malate dehydrogenase 2 (MDH2) enzyme and is involved in the oxidation of malate to oxaloacetate in the Krebs cycle (Calsina *et al.* 2018). Several other genes involved in metabolic pathways also have been implicated. Germline PVs in (i) the *DSLT* gene, which encodes a component of the multi-enzyme complex 2-oxoglutarate dehydrogenase (Buffet *et al.* 2021), (ii) the *SLC25A11* gene, whose product is the mitochondrial 2-oxoglutarate/malate carrier (Buffet *et al.* 2018), and (iii) the *GOT2* gene, which encodes the enzyme glutamic-oxaloacetic transaminase (Remacha *et al.* 2017), have all been implicated as possible susceptibility genes for PCC/PGL. In addition, the *DNMT3A* gene, whose product is involved in DNA methylation, has been identified as a potential susceptibility gene as well (Remacha *et al.* 2018). More work needs to be done to prove causality in many cases.

### Pediatric pheochromocytoma and paraganglioma

The estimated incidence of pediatric PCC/PGLs is 0.2–0.5 cases per million, with an estimated prevalence of 10–20% of all PCC/PGLs cases being diagnosed before the age of 18 (Bausch *et al.* 2014, Pamporaki *et al.* 2017). It is estimated that PCC/PGLs are the etiology for about 2% of primary pediatric hypertension cases (Wyszynska *et al.* 1992). Persistent hypertension (vs paroxysmal hypertension) is more commonly described as the presenting symptom in the pediatric versus adult population, although pediatric cases can present with paroxysmal hypertension as well (Barontini *et al.* 2006, Mishra *et al.* 2014). Pediatric patients are more often symptomatic at presentation with hypertension, palpitations, headache, and/or sweating occurring in greater than 85% of patients (Barontini *et al.* 2006, Bausch *et al.* 2014, Mishra *et al.* 2014, Park *et al.* 2020), and children can have atypical symptoms including abdominal pain and nausea. There are some phenotypic differences to note for pediatric vs adult PCC/PGL; the tumors in the pediatric population are more likely to be multifocal, metastatic (specifically nonsynchronous metastases), and recurrent (Pamporaki *et al.* 2017), highlighting the paramount importance of lifelong monitoring in children with PCC/PGLs.

Around 80% of pediatric PCC/PGLs are associated with hereditary germline PVs compared to 30–40% in the adult population (Bausch *et al.* 2014, Pamporaki *et al.* 2017). Pediatric cases are more likely to have cluster 1 susceptibility gene PVs, which likely explains the increased risk of recurrence and metastases associated with pediatric PCC/PGLs. Pediatric cohorts with PCC/PGL most commonly have germline PVs in *VHL* (range 30–50%) and *SDHB* (range 15–40%), whereas germline variants in *SDHD* (~10%), *NF1, RET, SDHC, SDHA,* and *MAX,* are much less commonly seen in the pediatric population (Cascón *et al.* 2013, Bausch *et al.* 2014, Babic *et al.* 2017, Pamporaki *et al.* 2017, de Tersant *et al.* 2020, Park *et al.* 2020). Risk estimates for metastatic PCC/PGL in the pediatric population vary from 10 to 50% (Bausch *et al.* 2014, Pamporaki *et al.* 2017, De Tersant *et al.* 2020). However, there is great variation in the length of follow-up in different studies, which makes exact estimates difficult.

Once PCC/PGL has been identified in a child, close monitoring and lifelong surveillance are recommended. Similar to the adult population, all pediatric patients with PCC/PGL should be offered clinical genetic testing, given the high hereditary component, and if positive, then the family should be offered cascade genetic testing so all affected family members can be screened. If cascade genetic testing identifies a pediatric PV carrier, screening varies based on the gene affected, in most cases, similar to that recommended for adults, as described in each gene section above and in Table 1. The risk of screening too frequently is the over-medicalization of children, and if CT or PET/CT scans are used instead of MRI, the risk of radiation becomes relevant. However, the risk of missing a new or recurrent tumor and/or metastatic disease in the pediatric population could also be life-limiting, so a balance must be struck.

## Conclusions and future perspectives

Knowledge of the germline genetics of PCC/PGL has significantly expanded over the past 20 years. It is now known that about 30–40% of patients with PCC/PGL and 80% of children with PCC/PGL carry a germline PV in one of over 12 well-defined susceptibility genes. Therefore, it is recommended that all patients who are diagnosed with PCC/PGL undergo clinical genetic testing. The phenotypes and recommended screening protocols are evolving as more data are collected over time. Importantly, no PCC/PGL can be considered fully benign and all patients should be followed for life for recurrence, new primary PCC/PGL, and metastatic disease.

## Declaration of interest

The authors declare that there is no conflict of interest that could be perceived as prejudicing the impartiality of this review.

## Funding

L F funded in part by NIH/NCI R01CA246586.

## Author contribution statement

The authors have contributed to the conceptualization and design, writing of original draft, review, editing, and approval of final version.
